# Supplementary material for: The cryptic gonadotropin-releasing hormone neuronal system of human basal ganglia
Source: eLife. 2021 Jun 15;10:e67714. doi: 10.7554/eLife.67714 (PMC8245125; doi:10.7554/eLife.67714)
Supplement: Supplementary file 1. — ChAT: choline acetyltransferase; ChINs: cholinergic interneurons; GW11: gestational week 11; IF: immunofluorescence; IHC: immunohistochemistry; ISH: in situ hybridization; HPLC-MS/MS: high-performance liquid chromatography-tandem mass spectrometry; PMI: postmortem interval; RIN: RNA integrity number; RNA-seq: RNA sequencing; SPNs: medium spiny projection neurons. [file elife-67714-supp1.docx]

| **ID** | **Sex** | **Age (years)** | **PMI** | **Brain region** | **Experiment** | |
| --- | --- | --- | --- | --- | --- | --- |
| #1 | F | 31 | <24 h | Striatum | IHC | GnRH cell counting |
| #2 | M | 61 | <24 h | Striatum | IHC | GnRH cell counting |
| #3 | M | 62 | <24 h | Striatum | IHC | GnRH cell counting |
|  |  |  |  |  | IF | GnRH/ChAT colocalization studies (Quantification of dual-labeled cells) |
|  |  |  |  | Hypothalamus | IF | GnRH/ChAT colocalization studies |
| #4 | F | 20 | <36 h | Striatum | IF | Dual-IF control; GnRH/ChAT colocalization studies |
| #5 | F | 37 | <24 h | Striatum | IHC | Positive control |
|  |  |  |  |  | IF | Dual-IF control; GnRH/ChAT colocalization studies |
| #6 | M | 62 | ~62 h | Striatum | IHC | Positive control |
|  |  |  |  | Hypothalamus | IF | GnRH/ChAT colocalization studies |
| #7 | F | 60 | ~24 h | Hypothalamus | IF | GnRH/ChAT colocalization studies |
| #8 | F | 84 | ~24 h | Hypothalamus | IF | GnRH/ChAT colocalization studies |
| #9 | F | 90 | <36 h | Hypothalamus | IF | GnRH/ChAT colocalization studies |
| #10 | M | 61 | ~24 h | Hypothalamus | IF | GnRH/ChAT colocalization studies |
| #11 | M | 79 | ~24 h | Hypothalamus | IF | GnRH/ChAT colocalization studies |
| #12 | F | 57 | <36 h | Striatum | IHC | Positive control |
| #13 | - | - | <36 h | Striatum | IHC | Positive control |
| #14 | M | 69 | <36 h | Striatum | IHC | Positive control |
| #15 | F | 77 | <24 h | Striatum | IHC | Positive control |
|  |  |  |  |  | ISH/IF | *GNRH1* mRNA detection in GnRH-IR cells |
| #16 | M | 56 | <36 h | Striatum | IHC | Positive control |
|  |  |  |  |  | ISH/IF | *GNRH1* mRNA detection in GnRH-IR cells |
| #17 | M | 49 | <24 h | Striatum | IHC | Positive control; Preabsorption control |
|  |  |  |  |  | ISH/IF | *GNRH1* mRNA detection in GnRH-IR cells |
| #18 | M | 63 | <36 h | Striatum | IHC | Positive control; Preabsorption control |
|  |  |  |  |  | ISH/IF | *GNRH1* mRNA detection in GnRH-IR cells |
| #19 | M | 64 | ~12 h | Striatum | IHC | Positive control; Preabsorption control |
|  |  |  |  |  | IF | GnRH/Chat colocalization studies |
|  |  |  |  |  | ISH/IF | *GNRH1* mRNA detection in GnRH-IR cells |
| #20 | F | 72 | ~24 h | Putamen | DiI | Studies of GnRH cell morphology |
| #21 | M | 82 | <6 h | Putamen | RNA-Seq | Transcriptome analysis of ChINs and SPNs (RIN: 5.7) |
|  |  |  |  |  | HPLC-MS/MS | Tissue GnRH/GnRH1-5 analytics |
| #22 | M | 91 | <24 h | Putamen | RNA-Seq | Transcriptome analysis of ChINs and SPNs (RIN: 4.1) |
|  |  |  |  |  | HPLC-MS/MS | Tissue GnRH/GnRH1-5 analytics |
| #23 | F | 70 | <6 h | Hypothalamus | HPLC-MS/MS | Tissue GnRH/GnRH1-5 analytics |
| #24 | M | 53 | <24 h | Hypothalamus | HPLC-MS/MS | Tissue GnRH/GnRH1-5 analytics |
| #25 | M | 66 | <24 h | Putamen | HPLC-MS/MS | Tissue GnRH/GnRH1-5 analytics |
|  |  |  |  | N. caudatus |  |  |
|  |  |  |  | Claustrum |  |  |
| #26 | M | 40 | <24 h | Putamen | HPLC-MS/MS | Tissue GnRH/GnRH1-5 analytics |
|  |  |  |  | N. caudatus |  |  |
| #27 | M | 59 | <24 h | Claustrum | HPLC-MS/MS | Tissue GnRH/GnRH1-5 analytics |
| #28 | F | 65 | <24 h | Putamen | HPLC-MS/MS | Tissue GnRH/GnRH1-5 analytics |
|  |  |  |  | Claustrum |  |  |
| #29 | N/A | GW11 | <3 h | Head | IF | GnRH/ChAT colocalization studies |
| #30 | N/A | GW11 | <3 h | Head | IF | GnRH/ChAT colocalization studies |
