# Supplementary material for: The cryptic gonadotropin-releasing hormone neuronal system of human basal ganglia
Source: eLife. 2021 Jun 15;10:e67714. doi: 10.7554/eLife.67714 (PMC8245125; doi:10.7554/eLife.67714)
Supplement: Supplementary file 2. — ChAT: choline acetyltransferase; GAP1: GnRH-associated peptide-1; GnRH: gonadotropin-releasing hormone; IF: immunofluorescence; IHC: immunohistochemistry; TSA: tyramide signal amplification. [file elife-67714-supp2.docx]

| **Primary Antibodies** | **Dilution & Application** | **Product code** | **Company/Source** | **Reference** |
| --- | --- | --- | --- | --- |
| rabbit, anti-GAP | 1:5,000 (IHC) | MC-2 | Produced by  Dr. Michael D. Culler | ([Culler et al., 1986](#_ENREF_8)) |
| rabbit, anti-GnRH | 1:10,000 (IHC) | LR-1 | Gift of Dr. Robert A. Benoit | ([Silverman et al., 1990](#_ENREF_45)) |
| guinea pig, anti-GnRH | 1:30,000 (IHC, IF-TSA);  1:10,000 (IF) | #1018 | Made in-house | ([Hrabovszky et al., 2011](#_ENREF_20)) |
| rat, anti-GnRH | 1:20,000 (IHC, IF-TSA) | #1044 | Made in-house | ([Skrapits et al., 2015](#_ENREF_46)) |
| sheep, anti-GnRH | 1:1,000 (IHC) | #2000 | Made in-house | ([Skrapits et al., 2015](#_ENREF_46)) |
| goat, anti-ChAT | 1:150 (IF); 1:2,000 (IF-TSA) | AB144P | Merck | ([Yonehara et al., 2011](#_ENREF_59)) |
|  |  |  |  |  |
| **Fluorescent reagents** | **Dilution** | **Product code** | **Company/Source** | **Reference** |
| FITC-tyramide; Cy3-tyramide | 1:1,000 | - | Synthesized in-house | ([Hopman et al., 1998](#_ENREF_19)) |
| donkey, AF488-anti-guinea pig IgG | 1:400 | 706-545-148 | Jackson ImmunoResearch |  |
| donkey, AF568-anti-goat IgG | 1:400 | A-11057 | Invitrogen |  |
|  |  |  |  |  |
| **Biotinylated reagents** | **Dilution** | **Product code** | **Company** |  |
| donkey, anti-guinea pig IgG | 1:500 | 706-065-148 | Jackson ImmunoResearch |  |
| donkey, anti-rabbit IgG | 1:500 | 711-065-152 | Jackson ImmunoResearch |  |
| donkey, anti-rat IgG | 1:500 | 712-065-153 | Jackson ImmunoResearch |  |
| donkey, anti-sheep IgG | 1:500 | 713-065-147 | Jackson ImmunoResearch |  |
| donkey, anti-goat IgG | 1:500 | 705-065-147 | Jackson ImmunoResearch |  |
|  |  |  |  |  |
| **Peroxidase-conjugated reagents** | **Dilution** | **Product code** | **Company** |  |
| donkey, anti-guinea pig IgG | 1:250 | 706-035-148 | Jackson ImmunoResearch |  |
| sheep, anti-digoxigenin; Fab fragments | 1:100 | 11207733910 | Roche |  |
| ABC Elite reagent | 1:1,000 | PK-6100 | Vector Laboratories |  |
